# Long-term prognosis of acute primary angle closure in an east asian cohort

**DOI:** 10.1007/s10384-024-01065-3

**Published:** 2024-05-13

**Authors:** Yoon Jeong, Ki Ho Park, Jin Wook Jeoung

**Affiliations:** Department of Ophthalmology, Seoul National University Hospital, Seoul National University College of Medicine, 101 Daehak-ro, Jongno-gu, Seoul, 03080 Republic of Korea

**Keywords:** Glaucoma, Angle-Closure, East Asia, Prognosis, Visual Fields

## Abstract

**Purpose:**

To provide an updated analysis of the long-term outcomes of patients with acute primary angle closure (APAC) and to investigate the risk factors for visual field (VF) loss progression.

**Study Design:**

Retrospective, clinical cohort study

**Methods:**

One hundred and forty-six APAC patients with a minimum of 1-year follow-up were included. The presenting features and the treatment utilized were recorded. The visual and intraocular pressure (IOP) outcomes were analyzed. The main outcome measures were the proportion of blindness and IOP at the final visit. A subset of patients with sufficient VF results was divided into a stable and progressive group based on mean deviation (MD) loss rate. Univariate and multivariate logistic regression analyses were performed to identify predictors of progression.

**Results:**

Nine patients (6.2%) were blind, and 76.0% (111/146) had final decimal visual acuity greater than or equal to 0.5. All patients had normal final IOP, and 65.1% (95/146) were medication-free. 64.4% (94/146) underwent cataract surgery at a median 4 months after their APAC attack. The use of topical hypotensive medications (OR = 8.029, P = 0.012) was the only significant predictor of fast MD loss in the multivariate regression.

**Conclusions:**

The long-term outcomes of APAC in recent years have been more promising. All patients maintained normal IOP several years following their APAC attack, and fewer than half required hypotensive agents. The incidence of blindness was low. These findings suggest that current practice patterns in the management of APAC are beneficial.

**Supplementary Information:**

The online version contains supplementary material available at 10.1007/s10384-024-01065-3.

## Introduction

Acute primary angle closure (APAC) is an important sight-threatening emergency, especially in Asia. The incidence rates of APAC in Asians are reported to be higher than in Caucasians: 59.95 cases per 1,00,000 person-years among Koreans, 114 cases per 1,00,000 person-years among Japanese, 122 cases per 1,00,000 person-years among Singapore Chinese, but only 25.0 per 1,00,000 person-years among Scots [1]. In addition to APAC’s being more prevalent in Asian eyes, it is also believed to be more severe in Asian eyes [2]. Studies with Caucasian subjects show that laser peripheral iridotomy (LPI) is effective in maintaining intraocular pressure (IOP) control over the long term after APAC [3–5]; however, among Asians, a high proportion (58.1%) of those with APAC developed increased IOP despite treatment by LPI [6]. Previously, a long-term outcome for Asians eyes with APAC was that one-fifth of APAC sufferers became blind, glaucoma being the cause in 50% [7]. Approximately 40% of those APAC patients who went blind had undergone previous filtering surgery [7].

The long-term visual prognosis after diagnosis of APAC in East Asian populations is still questionable [7–10] Over the recent decades, new APAC treatment modalities such as immediate argon laser peripheral iridoplasty (ALPI) or primary cataract extraction have emerged. Moreover, early phacoemulsification has appeared to be more effective in preventing IOP rise than LPI after an APAC attack [11]. Ha et al. report that the prevailing trend in APAC treatment changed between 2007 and 2017, patients now undergoing lens extraction more frequently [12].

This study aimed to investigate the long-term visual acuity (VA) and IOP outcomes in East Asian patients following an APAC attack. Its secondary objective was to identify any risk factors that may predict progression to more severe visual field (VF) loss. The findings of this study will provide valuable information on the visual outcomes and risk factors associated with APAC in East Asian populations.

## Methods

We retrospectively reviewed the medical records of all patients presenting to the Emergency Department of Seoul National University Hospital and diagnosed with APAC between June 2005 and April 2022. The study was conducted in accordance with the Declaration of Helsinki, and Institutional Review Board/Ethics Committee approval was obtained (No. 2207-156-1344).

### Study Participants

Patients were included in the study if they had presented with an IOP of more than 21 mmHg and had had at least one of the following symptoms—ocular or periocular pain, headache, nausea, vomiting, blurring of vision with haloes—and at least two of the following signs: conjunctival injection, corneal edema, mid-dilated pupil, shallow anterior chamber. Cases were excluded if they had been followed up for less than a year and/or showed any secondary causes of angle closure including lens-induced glaucoma, neovascular glaucoma, uveitis or aqueous misdirection syndrome.

Patient demographics and clinical features such as VA, IOP, axial length, duration of symptoms, time from presentation to definitive treatment, and type of treatment received were obtained. IOP at each visit was also recorded. IOP fluctuations were defined as a standard deviation of IOP measurement. Snellen VA was converted to LogMAR units preparatory to a statistical analysis. For non-numerical VA, the following denotations were used: finger counting = 1.7 LogMAR, hand movement = 2.0 LogMAR, light perception = 2.3 LogMAR, and no light perception (NLP) = 3.0 LogMAR. The main outcome measure was blindness, defined as best-corrected VA worse than logMAR 1.30.

For individuals who received LPI and did not require additional intraocular surgery to resolve angle closure, their endothelial cell count (ECC) after the LPI was collected. To ensure sufficient time for corneal edema to resolve after the APAC and LPI, only the cases where ECC was measured at least 1 month after LPI were included [13]. The measurements were taken using noncontact specular microscopes (Noncon ROBO SP-8800 and KONAN CellChek 20 before and after May 1, 2021, respectively; Konan Medical).

### Subgroup Analysis with Reliable Visual Field Tests

For the patients who had undergone standard automated perimetry (SAP), its values were collected when they were reliable, defined as those with a fixation loss rate ≤ 20%, a false-positive rate ≤ 15%, and a false-negative rate ≤ 25%. All SAPs were performed using the 24-2 program of the Humphrey Visual Field Analyzer (Zeiss Inc.) with the standard Swedish interactive threshold algorithm. For a subgroup of patients who had undergone at least 3 or more reliable SAPs, linear regression analyses of mean deviation (MD) were carried out against time, and the corresponding regression slope (in dB per year) was calculated. We categorized the patients into stable and progressive groups based on a cutoff of−0.5dB/y.

### Time-Stratified Analysis

Since a variety of cases may be mixed due to the wide period covered, (between June 2005 and April 2022), we conducted additional data analysis to find potential alterations in long-term outcomes or treatment practices over the extended study period. We categorized the patients into two groups based on the year 2017 as a reference point. We selected 2017 following the publication of the EAGLE trials in October 2016, which emphasized the importance of clear lens extraction in angle-closure glaucoma [14].

### Statistical Analysis

One eye of each subject, randomly selected in bilateral cases, or the affected eye in unilateral cases, was selected for the statistical analysis. The data were analyzed using an unpaired t test, Mann-Whitney test, and Fisher’s exact probability test. Univariate and multivariate logistic regression analyses were run to find predictors of VF progression. Variables with a *P* value less than 0.1 in the univariate analysis were included in the multivariate model. A *P* value of less than 0.05 was considered statistically significant. All the statistical analyses were performed with SPSS software version 27 (SPSS Inc.).

## Results

### Study Subjects

A total of 190 APAC patients visited the Emergency Department of Seoul National University Hospital between June 2005 and April 2022. Among them, 146 were eligible for the study. Table 1 shows the baseline demographic data for the cohort. The male-to-female ratio was 1:3.4. At presentation, the patients were 64.8±8.2 years old with VA of LogMAR 1.0±0.7 and IOP of 50.7±11.3 mmHg. The acute attack affected both eyes simultaneously in eleven patients (7.5%). Seven (4.8%) had already received LPI from an outside hospital in the affected eye. Eighty-six patients (58.9%) presented to the hospital within 24 h of symptom onset. There were 10 cases that experienced prolonged attacks before seeking medical attention. Among them, 5 cases had attacks lasting over 7 days, and another 5 cases had attacks lasting over 14 days.

**Table 1:** Baseline patient demographics (N=146)

	N (%)
Male: female ratio	1:3.4
Age, years	64.8±8.2
Presenting IOP, mmHg	50.7±11.3
Presenting VA, logMAR	1.0±0.7
Duration of symptoms	
<24 h	86 (58.9)
24–72 hr	35 (24.0)
>72 hr	25 (17.1)
Mean ± SD, h	75.5±247.1
Axial length, mm	22.6±0.9
Previously received LPI	7 (4.8)
Simultaneous bilateral attack	11 (7.5)

*IOP* intraocular pressure, *LPI* laser peripheral iridotomy, *SD* standard deviation, *VA* visual acuity

**Fig. 1:**
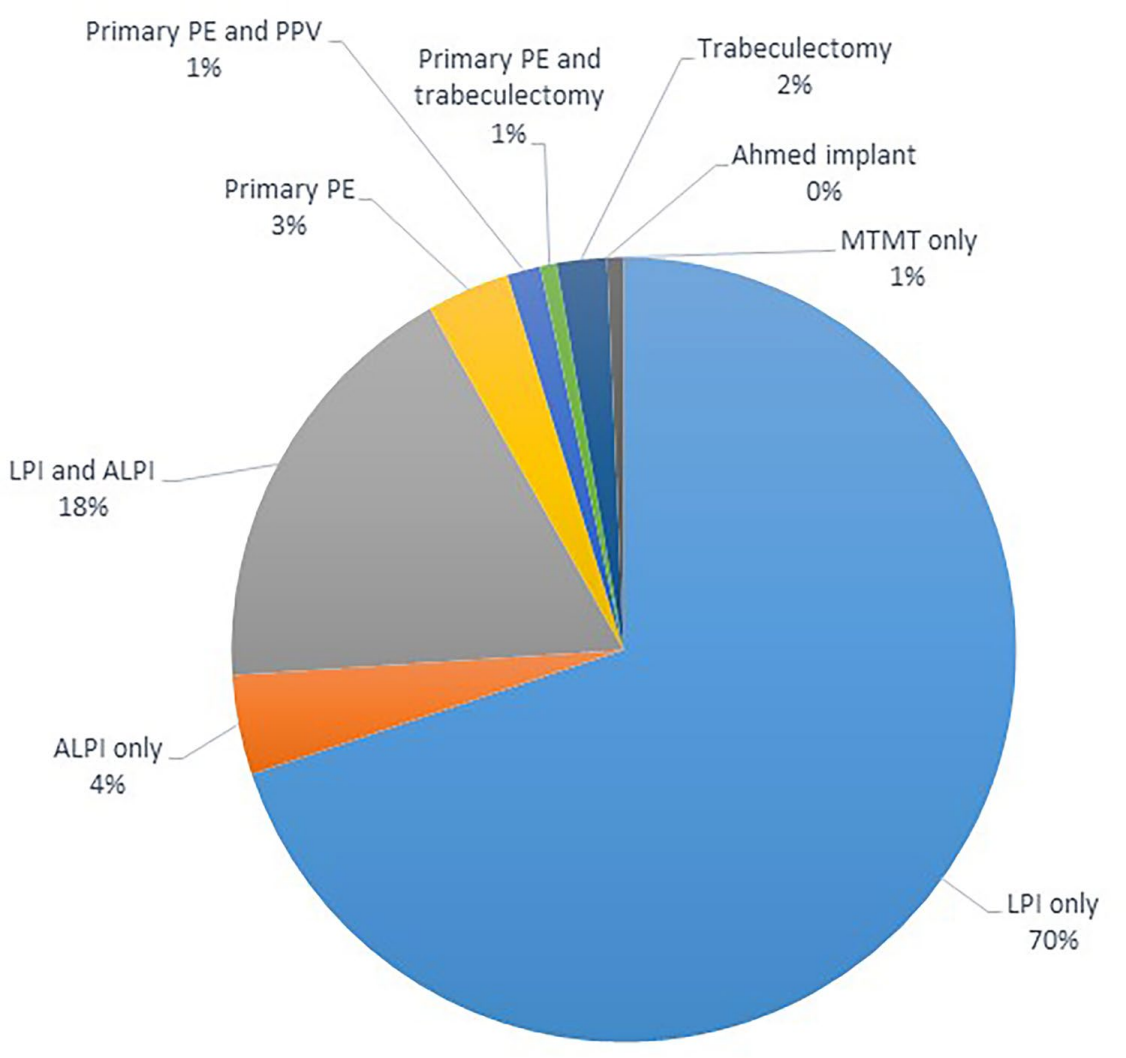
Primary treatment modalities for acute primary angle closure (APAC) attack. 134 patients (91.8%) underwent primary laser treatment (laser peripheral iridotomy and/or laser iridoplasty) only, while 11 patients (7.5%) required ocular surgery. The most frequent surgical option was primary phacoemulsification

Details on the primary treatment modalities are shown in Fig. 1. Regarding the mode of treatment, the cases of 134 patients (91.8%) were resolved with laser treatment (LPI, ALPI, or both) only, while eleven patients (7.5%) required ocular surgery. The most frequent surgical option was primary phacoemulsification.

### Outcomes at Final Visit

The patients were followed-up for a mean period of 5.4±4.1 years (range, 1.0–16.1 years). Table 2 lists the clinical outcomes at their final visits. Initially, 78 of 146 patients (53.4%) had presenting VA of less than 0.1, but at the final visit, 136 patients (93.1%) had final VA better than or equal to 0.1, and 76.0% (111 of 146) had final VA greater than or equal to 0.5. Nine patients (6.2%) became blind. The causes of blindness were bullous keratopathy (3 patients), cataract (2 patients), glaucomatous optic neuropathy (2 patients), and retinal disease (2 patients). All patients had normal IOP at the final visit, and the mean final IOP was 13.02 mmHg. Fifty-one patients (34.9%) were on at least one topical IOP-lowering medication, and 95 patients (65.1%) were medication-free at the final visit. Among the 51 patients on topical IOP-lowering medications, 16 (11.0%) were on a single agent, 24 (16.4%) were on two, 9 (6.2%) were on three, and 2 (1.4%) were on four.

**Table 2:** Clinical outcomes at the last follow-up (N=146)

	N (%)
Duration of follow-up, years	5.4±4.1
VA	
<0.05 (blind)	9 (6.2)
0.05≤VA< 0.1 (severe visual impairment)	1 (0.7)
0.1≤VA**<**0.5	25 (17.1)
0.5≤VA<0.8	54 (37.0)
≥0.8	57 (39.0)
Mean ± SD, logMAR	0.3±0.5
IOP	
>21 mmHg	0 (0)
15–21 mmHg	38 (26.0)
<15 mmHg	108 (74.0)
Mean ± SD, mmHg	13.0±2.6
On topical hypotensive agents	51 (34.9)
Single substance	16 (11.0)
Two substances	24 (16.4)
Three substances	9 (6.2)
Four substances	2 (1.4)
Recurrence of attack in the same eye	6 (4.1)
Subsequent attack in the opposite eye	4 (2.7)

*IOP* intraocular pressure, *SD* standard deviation, *VA* visual acuity

By the end of their last visit, six patients (4.1%) had experienced a repeated attack in the same eye, and four patients (2.7%) a subsequent attack in the opposite eye, despite prophylactic LPI. In fact, among 17 patients who experienced repeated attack even after prior LPI, nine (52.9%) required the re-opening of their laser holes due to a non-patent LPI site. Over the course of follow-up, 94 patients (64.4%) underwent cataract surgery a median 4 months after their APAC attack; 80 (85.1%) underwent simple cataract surgery, while 11 underwent combined surgery with vitrectomy and 3, combined surgery with either trabeculectomy or Ahmed implant surgery. In total, 15 patients (10.3%) had glaucoma surgery: 8 patients trabeculectomy, 6 Ahmed implant surgery, and one, both trabeculectomy and Ahmed implant surgery. Out of the eleven participants who underwent vitrectomy, three received full vitrectomy, seven had core vitrectomy, and one underwent anterior vitrectomy.

Among the patients who received LPI and did not require additional intraocular surgery to resolve angle closure, there were 70 cases where ECC was measured at least 1 month after LPI. These 70 individuals had their ECC measured on average 28.6 ± 35.0 months (range 31 days–12.4 years) after LPI, with an average ECC value of 2491.9 ± 548.0 (range 425–3225). Only 4 (5.7%) patients had ECC values lower than 1500.

### Subgroup Analysis with Reliable Visual Field Results

Fifty-four (54) of 146 patients (40.0%) had at least three or more reliable SAPs for the subgroup analysis. The subgroup demographics are summarized in Table 3. Each patient took approximately six VF tests, and the overall baseline MD was −6.83±7.17 dB at a mean age of 65.67±7.04 years. The mean rate of MD loss was −0.39 dB/year (SD, 1.19) during a mean follow-up period of 5.95±3.47 years. Forty-one patients underwent cataract surgery and 13 did not undergo cataract surgery during the follow-up period. The patients who did not undergo cataract surgery displayed a faster VF progression than those who received cataract surgery (−0.79±1.39 dB/year vs. −0.26±1.11 dB/year, respectively), but the difference did not reach statistical significance (p = 0.225).

**Table 3:** Subgroup analysis with SAP (N=54)

	N (%)
Number of tests, n	5.6±2.6
Duration of follow-up, years	6.0±3.5
Baseline age, years	65.7±7.0
Baseline MD, dB	−6.8±7.2
Final MD, dB	−8.7±9.6
MD change	
>−0.5 dB/year (stable group)	39 (72.2)
≤−0.5 dB/year (progressors)	15 (27.8)
Mean±SD, dB/year	−0.4±1.2

*MD* mean deviation, *SD* standard deviation, *SAP* standard automated perimetry

**Fig. 2:**
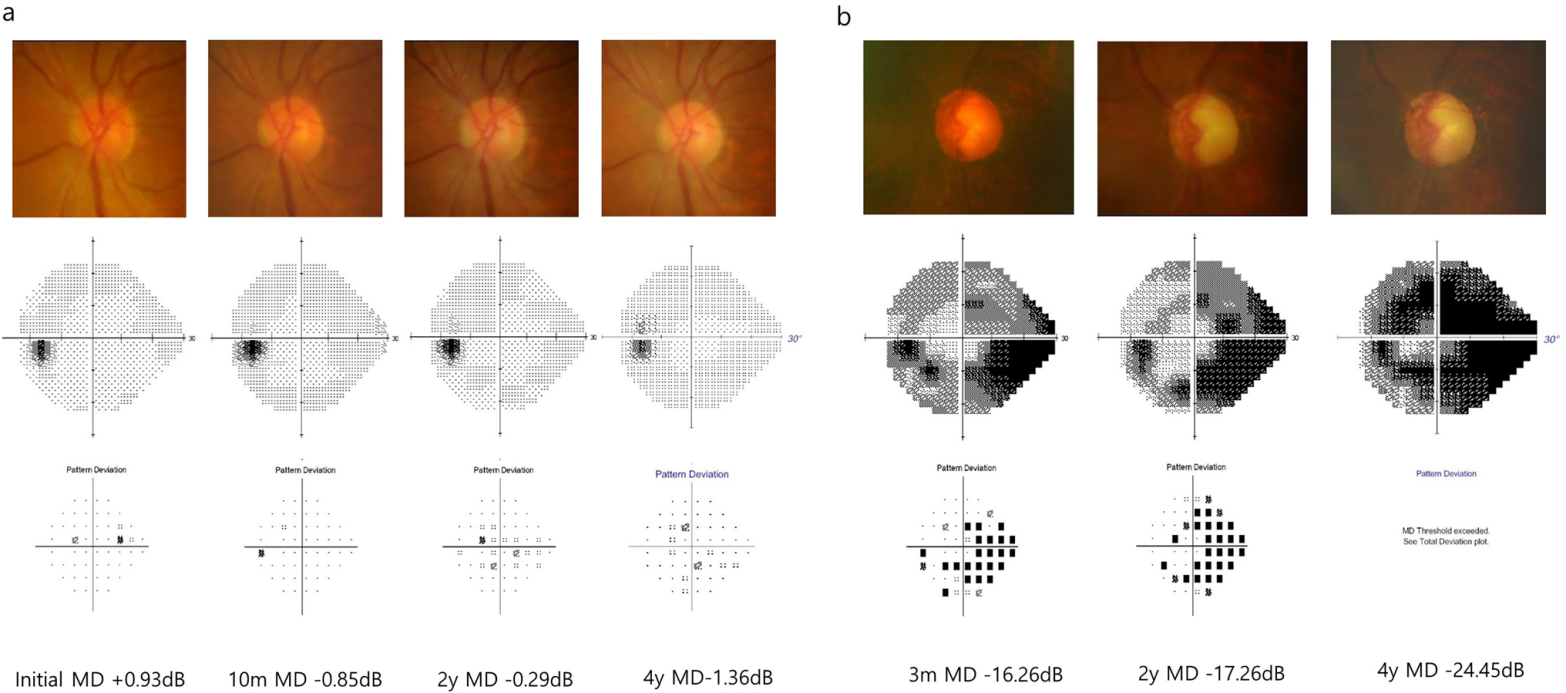
Representative cases: **a** stable patient, **b** progressive patient. **a** A 58-year-old female showed a stable prognosis for more than four years after the acute primary angle closure (APAC) attack. Presenting visual acuity (VA) was finger count, and the final VA was 0.5. She maintained normal intraocular pressure (IOP) (11 mmHg) without any topical hypotensive agent. Mean annual mean deviation (MD) loss was −0.389 dB/year. **b** A 65-year-old male showed fast progression for 4 years after the APAC attack. Presenting VA was 0.5, and the final VA was 0.2. He relied on 2 topical hypotensive agents to maintain normal IOP (11 mmHg). The mean annual MD loss was −2.271 dB/year

Among the 54 patients, 15 (27.8%) were progressors (≤−0.5dB/yr) and 39 (72.2%) were stable (>−0.5dB/yr). Representative cases, one stable patient and one progressive patient each are highlighted in Fig. 2a and b, respectively. Table 4 compares the demographic characteristics, biometric data, SAP information, and surgical history between the two groups (stable vs. progressors). Neither the symptom duration nor the time taken to resolve the APAC attack were significantly different between the two groups. The initial treatment types were comparable between the two groups, except that the progressive group had a higher number of cases that received ALPI only. The proportion of patients who underwent cataract surgery during the follow-up was similar between the two groups, and the time taken until cataract surgery also did not show any difference. Both groups had normal final IOP, but the progressive group underwent more IOP fluctuation over the course of follow-up (2.8mmHg vs. 4.3mmHg, *P*=0.087). Also, a greater portion of patients in the progressive group were on IOP-lowering medication than in the stable group (86.7% vs. 43.6%, *P*=0.004).

**Table 4:** Comparison of patient characteristics between stable group and progressive group

	Stable group (N=39)	Progression group (N=15)	*P* value
Initial			
Baseline age, years	64.5 ± 6.5	64.3±8.2	0.933
Axial length, mm	22.5 ± 0.7	22.6±0.7	0.685
Initial VA, logMAR	1.2 ± 0.6	0.7±0.7	0.032
Initial IOP, mmHg	53.2±11.8	44.2±11.0	0.014
Duration of symptoms, days	3.8±10.7	2.4±2.8	0.624
Time taken to IOP normalization, days	2.6±9.2	1.2±3.7	0.239
Baseline SAP MD, dB	−5.6±6.3	−10.1±8.4	0.035
Initial treatment type			
LPI only	25 (64.1%)	8 (53.3%)	0.467
ALPI only	1 (2.6%)	3 (20.0%)	0.060
LPI + ALPI	10 (25.6%)	3 (20.0%)	1.000
Primary PE	1 (2.6%)	0 (0%)	1.000
Trabeculectomy	1 (2.6%)	1 (6.7%)	0.482
MTMT only	1 (2.6%)	0 (0%)	1.000
During the follow-up			
Duration of follow-up, years	8.3±4.4	6.9±4.1	0.314
Average IOP, mmHg	13.4±2.1	14.4±3.0	0.239
IOP fluctuation, mmHg	2.8±1.8	4.3±2.8	0.087
Resolved by LASER only	37 (94.9%)	14 (93.3%)	1.000
Received glaucoma surgery	4 (10.3%)	4 (26.7%)	0.197
Received cataract extraction	32 (82.1%)	9 (60.0%)	0.152
Time taken until cataract surgery (months)	37.5±49.7	16.1±32.5	0.141
Final			
Last VA, logMAR	0.2±0.2	0.6±0.5	0.007
Last IOP, mmHg	12.6±2.0	12.8±2.4	0.977
Last vertical CDR	0.6±0.1	0.8±0.12	0.000
On topical hypotensive agents	17 (43.6%)	13 (86.7%)	0.004
Final SAP MD, dB	−5.1±6.6	−18.3±9.8	0.000
MD slope, dB/year	0.2±0.4	−1.9±1.2	0.000

*CDR* cup-to-disc ratio, *IOP* intraocular pressure, *LPI* laser peripheral iridotomy, *ALPI* argon laser peripheral iridoplasty, *PE* phacoemulsification, *MTMT* maximal tolerable medical therapy, *MD* mean deviation, *SAP* standard automated perimetry, *VA* visual acuity

According to the univariate analysis, the IOP fluctuation during the follow-up (odds ratio [OR] = 1.319, *P* = 0.042) and the use of anti-glaucoma medication (OR = 8.412, *P* = 0.010) were significant predictors of faster MD loss (Table 5). IOP fluctuation, baseline SAP MD values, and the use of anti-glaucoma medication all had *P* values less than 0.1 and, thus, were included in the subsequent multivariate regression analysis. The use of anti-glaucoma medication (OR = 8.029, *P* = 0.012) was the only significant predictor of greater MD loss in both the univariate and multivariate regression analyses.

**Table 5:** Predictors of VF MD loss after acute angle closure

	Univariate logistic regression analysis			Multivariate logistic regression analysis		
	Odds ratio	95% CI	*P* value	Odds ratio	95% CI	*P* value
Baseline age, years	0.992	0.910–1.081	0.854			
Sex	0.438	0.113–1.687	0.230			
Duration of symptoms, days	0.999	0.995–1.003	0.624			
Time taken from presentation to IOP normalization, hours	0.999	0.996–1.002	0.448			
IOP average, mmHg	1.191	0.920–1.543	0.184			
IOP fluctuation	1.319	1.010–1.723	0.042^*^			
Last IOP, mmHg	1.025	0.771–1.364	0.864			
On topical hypotensive agents	8.412	1.669–42.406	0.010^*^	8.029	1.589–40.582	0.012
Baseline SAP MD, dB	0.923	0.849-–1.004	0.063			

*CI* confidence interval, *IOP* intraocular pressure, *MD* mean deviation, *SAP* standard automated perimetry, *VF* visual field

### Time-Stratified Analysis

The analysis results, comparing the two groups based on the year 2017, are presented in Supplement Table 1 and 2. The long-term outcomes such as final VA, IOP, or the use of IOP-lowering medications remained comparable between the two groups. The frequency of immediate primary lens extraction also appeared similar, but there was a noticeable trend toward earlier timing of cataract surgery. Before 2017, cataract surgery was performed on average 31.5 months after the attack, whereas in recent times, it occurred much earlier, with an average of 5.9 months post-attack.

Among 54 patients with reliable VF test results, the recent group (after 2017) showed a slower VF deterioration at a rate of −0.33 dB/year, while the early group (before 2017) exhibited a deterioration at a rate of −0.41 dB/year. However, the difference in the MD slope between the two groups did not reach statistical significance.

## Discussion

The findings of this study indicate an improvement in the long-term outcomes of patients with APAC compared to previous reports. In three earlier studies covering the 1990s to the early 2000s, 41.8–50% of patients had IOP < 21 mmHg without medication, 13.8–32.7% required additional glaucoma surgery, and 58% had poor VA worse than 6/9 [6–8]. On the other hand, all subjects in our study showed IOP controlled to < 21 mmHg at their final visit, 65.0% being medication-free, and 10.3% received glaucoma surgery (trabeculectomy, glaucoma drainage device implant, or both) for IOP control. Ninety-seven (97) patients (66.4%) had VA of 0.6 or better at the final visit, and only 9 patients were blind.

The use of primary laser treatments, especially ALPI, appears to have contributed to these positive outcomes. In the current study, 22% of patients received ALPI, whereas in older studies prior to the widespread application of ALPI, subjects mainly received LPI, with fewer receiving ALPI [6, 8] ALPI has been proposed as a means of increasing the angle width and reducing the subsequent development of peripheral anterior synechiae (PAS) [15] Since PAS extent is one indicator of the risk of developing subsequent uncontrolled IOP after elimination of the relative pupillary block [9], the more frequent use of ALPI in our study may have resulted in a lower percentage of patients requiring glaucoma medication or surgery.

Furthermore, the noteworthy trend toward earlier timing of cataract surgery in our study implies that the more favorable outcomes observed in our study, compared to earlier research, may be associated with the practice of performing cataract surgery at an earlier stage. Individuals with APAC may have a high prevalence of cataracts at the time of their attack, for lenses thicken and move relatively more anteriorly with aging. As the cataract itself provides media opacity and decreases patients’ VA, early cataract extraction may improve visual outcomes in APAC patients. Also, since cataractous lenses may play a role in the pathogenesis of angle closure [16, 17], prompt removal of such abnormally anteriorly positioned lenses may prevent persistent IOP elevation in cases of shallow anterior chamber and chronic angle closure after APAC. In fact, the patients in our study who did not undergo cataract surgery displayed slightly faster VF progression than those who received cataract surgery.

Although the prognosis was generally favorable, 27.8% of the subjects experienced a rapid loss in their VF. In the multivariate regression analysis, the use of topical hypotensive medication was found to be a significant predictor of fast MD loss. The underlying mechanisms remain unclear, but it is possible that clinicians have added hypotensive agents to the treatment regimens of patients whose IOP control had been unstable, since our progression group showed a greater tendency to IOP fluctuation. Even if both stable and progressive groups show similar mean IOP, possibly due to the use of hypotensive agents, optic nerve damage may yet occur and accumulate during IOP fluctuation periods.

Given the typical correlation between the extent of PAS and IOP control in primary angle closure glaucoma (PACG), it is important to acknowledge the potential contribution of PAS to IOP fluctuations and the use of glaucoma medication in our patients. However, the extent of PAS in patients was not confirmed, so we cannot be certain about its association with IOP fluctuation. Exploring the extent of PAS in future prospective studies could enhance our understanding of this relationship. Regardless, monitoring APAC patients at shorter intervals may be necessary to ensure that IOP spikes do not go untreated for too long.

An interesting finding from our study is that a total of 17 patients (11.6%) experienced a second APAC attack, even after treatment by LPI. Intriguingly, half of these patients still had a patent LPI. In contrast to other studies indicating much lower recurrence rates approaching zero [6, 8, 9], our findings suggest that LPI may not uniformly succeed in widening the angle in all APAC cases. The distinctive anatomical characteristics of Koreans, specifically a smaller anterior chamber and a thicker iris compared to the Chinese population [18], may contribute to a more intricate form of APAC beyond a simple pupillary block. It is plausible that LPI was ineffective in opening the angle for eyes predominantly characterized by a plateau iris configuration. Previous research by Xu et al. identified a flatter iris curvature as a risk factor for the progression from primary angle-closure suspect to either primary angle-closure (PAC) or APAC [19], potentially explaining the inconsistent benefits of LPI across all cases.

Moreover, among those who experienced repeated attacks post-LPI, half exhibited a non-patent LPI site. This observation could imply that, apart from the plateau iris configuration, the long-standing effect of LPI is less effective in Koreans who have thicker irises. An intriguing case report even documents a Korean patient with LPI obstruction occurring up to four times [20]. Cataract surgery can also widen the anterior chamber angle effectively in primary angle closure disease [21]. However, the efficacy of primary lens extraction may be insufficient in cases involving a thick iris with a plateau iris configuration. If angle closure attacks persist post-LPI or the iris appears thick, considering an initial strategy involving slightly larger than conventional LPI sites or actively expanding the size during the follow-up period, especially if the LPI site appears to be diminishing, could prove advantageous. Additional interventions like ALPI may be considered to physically alter the configuration of the peripheral iris. There is also an indication that endoscopic cyclophotocoagulation combined with cataract extraction could be beneficial, and exploring such treatments may be worthwhile [22].

There are several limitations to this study. It was retrospective, with variable follow-up periods, and involved multiple ophthalmologists in the care of the patients. There may be a potential mixing of various cases due to the broad period covered, ranging from 2005 to 2022. Nevertheless, we verified that the diversity of cases was comparable despite the extensive study duration. The long-term outcomes and the percentage of patients undergoing immediate primary lens extraction showed similarity between the two time periods.

Also, some may question why only a few cases opted for cataract surgery as the initial treatment in our study even if primary lens extraction has become a prevalent treatment for APAC [14]. Despite the potential benefits of clear lens extraction in regions with limited healthcare resources, where access to medications and monitoring may pose challenges, South Korea is well-equipped with highly accessible healthcare resources. Traverso has highlighted technical challenges and ongoing progressive endothelial cell loss associated with phacoemulsification for PAC [23]. He underscores that the justification for employing clear lens extraction in treating all patients with PAC, with or without glaucoma, is not yet conclusive. To mitigate potential risks, especially for inflamed eyes, we continue to address PAC events with LPI or ALPI rather than opting for immediate cataract surgery. Despite this cautious approach, the long-term outcomes after APAC were favorable in our study. We tentatively propose that initiating treatment with LPI and subsequently proceeding to early cataract surgery more judiciously may be an acceptable strategy for achieving positive long-term outcomes.

These facts notwithstanding, this study is notable for its distinctive approach to the analysis of the VF loss-progression rate in patients after an APAC attack. Most evaluations of glaucoma progression with VF loss have used data from open-angle glaucoma patients [24–27], and only few studies report VF changes in PACG patients, with relatively small samples [28–31]. We discovered that the overall rate of MD loss in APAC patients was −0.39 dB/year in this study, and 72.2% showed a stable course with annual MD loss less than 0.5 dB. This rate of deterioration was relative faster than in previous studies on VF loss progression in PACG patients, which reported an MD loss of −0.12 to −0.32 dB/year [28, 30, 31]. Such differences may have arisen from the fact that our study included only APAC patients, whereas the others included all PACG patients. However, since the proportion of patients with adequate VF test results was only 40.0%, it is difficult to generalize based on our results alone. A prospective large-scale, intensive investigation is required to identify any risk factors for poor prognosis or treatment that may improve the prognosis.

In conclusion, the long-term outcomes of APAC in recent years have been more favorable than in the past, though a small proportion of patients indeed show fast progression. This study may provide valuable insights into the long-term outcomes of APAC that highlight the importance of careful management and close monitoring of VF function in patients. Further studies are needed to better understand the factors contributing to VF loss progression in APAC patients and to develop more effective management strategies to prevent or slow down this progression.

### Supplementary Information

Below is the link to the electronic supplementary material.

Supplementary file1 (DOCX 15 KB)
