# Supplementary material for: Long-term prognosis of acute primary angle closure in an east asian cohort
Source: Jpn J Ophthalmol. 2024 May 13;68(4):302–10. doi: 10.1007/s10384-024-01065-3 (PMC11349775; doi:10.1007/s10384-024-01065-3)
Supplement: Supplementary file 1 — Supplementary file1 (DOCX 15 KB) [file 10384_2024_1065_MOESM1_ESM.docx]

**Supplement table 1. Time-stratified analysis of all subjects**

|  | **Early (N=87)** | **Recent (N=59)** | ***P* value** |
| --- | --- | --- | --- |
| **Final VA (logMAR)** | 0.36±0.51 | 0.28±0.38 | 0.345 |
| **Final IOP (mmHg)** | 12.77±2.46 | 13.32±2.66 | 0.200 |
| **On medication** | 28 (32.2%) | 23 (39.0%) | 0.480 |
| **Received cataract surgery as primary treatment** | 3 (3.4%) | 5 (8.5%) | 0.269 |
| **Eventually received cataract surgery** | 58 (66.7%) | 36 (61.0%) | 0.484 |
| **Time taken until cataract surgery (months)** | 31.52±44.48  (0.03-179) | 5.94±9.20  (0.03-39.23) | 0.001 |

The early group covers 2005.6.1 ~ 2017.1.1 and the recent group covers 2017.1.1 ~ 2020.12.

VA: visual acuity; IOP: intraocular pressure

**Supplement table 2. Time-stratified analysis of patients with reliable visual field tests**

|  | **Early (N=40)** | **Recent (N=14)** | ***P* value** |
| --- | --- | --- | --- |
| **MD slope (dB/yr)** | -0.41 | -0.33 | 0.828 |
| **Proportion of progressor** | 10 (25.0%) | 5 (35.7%) | 0.498 |

The early group covers 2005.6.1 ~ 2017.1.1 and the recent group covers 2017.1.1 ~ 2020.12.

MD: mean deviation
